# Light Emission Properties of a Cross-Conjugated Fluorene Polymer: Demonstration of Its Use in Electro-Luminescence and Lasing Devices

**DOI:** 10.3390/polym8020043

**Published:** 2016-02-05

**Authors:** Sergio Romero-Servin, Luis-Abraham Lozano-Hernández, José-Luis Maldonado, Ramón Carriles, Gabriel Ramos-Ortíz, Enrique Pérez-Gutiérrez, Ullrich Scherf, Mikhail G. Zolotukhin

**Affiliations:** 1 Centro de Investigaciones en Óptica A. P. 1-948, 37150 León Guanajuato, Mexico; sromero@cio.mx (S.R.-S.); luis_abraham90@hotmail.com (L.-A.L.-H.); eperez@cio.mx (E.P.-G.); 2 Macromolecular Chemistry Group, Wuppertal University, Gauss-Str. 20, D-42097 Wuppertal, Germany; scherf@uni-wupertal.de; 3 Instituto de Investigaciones en Materiales, Universidad Nacional Autónoma de México, A. P. 70-360, 04510 México D. F., Mexico; zolotukhin@iim.unam.mx

**Keywords:** fluorene cross-conjugated polymer, polymer light emitting diodes (PLEDs), lasing properties

## Abstract

Light emission properties of a fluorene cross-conjugated polymer (**PF–1**) based on the monomer 4,7-bis[2-(9,9-dimethyl)fluorenyl] benzo[1,2,5]thiadiazole are reported. This polymer exhibits solubility at high concentrations, good processability into thin solid films of good quality and a broad emission band with a fluorescence quantum yield of approximately 1. Based on these features, in this paper we implemented the use of **PF–1** as an active layer in polymer light-emitting diodes (PLEDs) and as a laser gain medium in solution. To get insight on the conducting properties of **PF–1**, two different electron injectors, poly [(9,9-bis(3′-(*N*,*N*-dimethylamino) propyl)-2,7-fluorene)-alt-2,7-(9,9–dioctylfluorene)] (**PFN**) and lithium fluoride (**LiF**), were used in a simple PLED architecture. PLEDs with the **PFN** film were found to exhibit better performance with a maximum luminous efficiency of 40 cd/A, a turn-on voltage (*V*_on_) of approximately 4.5 V and a luminance maximum of 878 cd/m^2^ at 5.5 V, with a current density of 20 A/m^2^. For the lasing properties of **PF–1**, we found a lasing threshold of around 75 μJ and a tunability of 20 nm. These values are comparable with those of rhodamine 6G, a well-known laser dye.

## 1. Introduction

Since the first report of electroluminescence in conjugated polymers in 1990 [1], numerous materials of this kind have been designed and synthesized to be used as active compounds for polymer light-emitting diodes (PLEDs). Over the last two decades great progress has been made in these materials reaching internal quantum efficiencies of approximately 100% [2,3]. PLEDs have several advantages with respect to solid-state inorganic semiconductors, such as flexibility, fast processing and ease of deposition in thin film**s** with good morphology by simple techniques such as spin-coating [4]. Nowadays, PLEDs and OLEDs (Light-Emitting diodes made of organic molecules of low molecular weight) are widely used in flat panel displays of several devices such as digital cameras, mobile phones and televisions [5]. Nevertheless, two of the challenges faced by the field of electro-luminance devices are to improve the manufacturing process including deposition methods, and the design of novel chemical structures with high fluorescence quantum yield which increases the emission spectrum and current-voltage characteristics [6,7,8,9]. The use of highly fluorescent materials, such as oligomers and polymers, opens the possibility of improving the PLEDs’ performance [8,10]. Among the wide variety of materials used for PLEDs, fluorene-based polymers show interesting and unique chemical and physical properties such as a rigid planar biphenyl unit, which improves solubility, as well as thermal stability and processability [10,11,12]; they comprise aromatic segments which help to reduce the turn-on voltage (*V*_on_) that results from a good balance in electron and hole injection [13,14].

**Figure 1:**
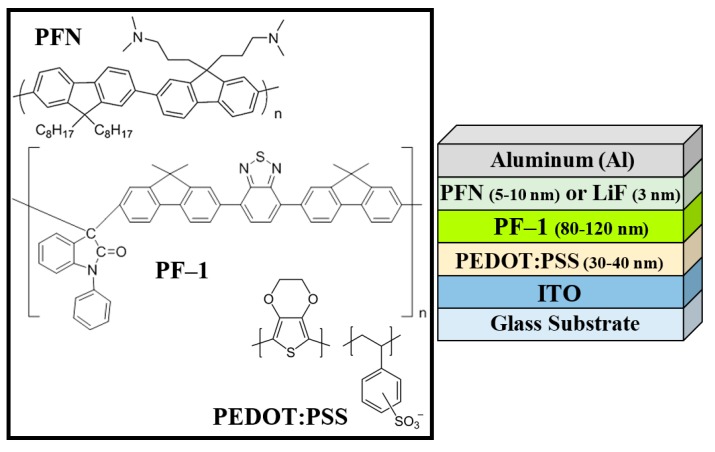
Chemical structure of **PFN**, conjugated polymer **PF–1**, and **PEDOT:PSS**; and PLEDs architecture.

Fluorene-based polymers are also under active research for other applications such as: organic photovoltaic (OPV) cells [15,16], two- and three-photon absorption phenomena [17,18,19,20], multiphoton fluorescence and imaging [21], two-photon micro-fabrication [22,23], optical power limiting [24,25] and optical data storage [26]. On the other hand, cross-conjugated compounds are relatively unexplored as active materials in optoelectronic or photonic devices; they can be found as quinones, radialenes, fulvalenes and fused aromatics [27]. Cross-conjugated systems have been implemented as PLEDs [28], nonlinear optical materials, and magnetic materials, and they also favor donor-acceptor interactions [29,30,31,32]. When the cross-conjugated polymer chain is connected to a donor material, a strong intramolecular charge transfer is observed, leading to high fluorescence intensities [30]. Motivated by this emission property, our group had previously synthesized the fluorene cross-conjugated polymer (**PF–1**), whose chemical structure is shown in Figure 1, and used this material to develop organic nanoparticles which exhibit very intense fluorescence induced by two-photon absorption in the wavelength range between 740 and 820 nm; these advantageous properties of nanoparticles of **PF–1** were used to implement their use in multiphoton microscopy [31]. Likewise, we demonstrated coherent frequency conversion within the telecommunication wavelength band (1100–1600 nm) [32].

Based on the above discussion of fluorene polymers and cross-conjugated compounds, and motivated by the excellent properties of **PF–1** (intense and broad emission band, solubility at high concentrations and easy processability into thin solid films of good quality), in this work we study the photophysical and electroluminescence characteristics of **PF–1** as an active material in an optoelectronic application (PLEDs) and in lasing. The conducting characteristics of this cross-conjugated polymer were studied with a simple PLED architecture in which a thin active layer of **PF–1** was sandwiched between the hole injector sheet poly(2,3-dihydrothieno-1,4-dioxin)-poly(styrenesulfonate) (**PEDOT:PSS**) and the electron injector films poly[(9,9-bis(3′-(*N*,*N*-dimethylamino)propyl)-2,7-fluorene)-alt-2,7-(9,9–dioctylfluorene)] (**PFN**) or lithium fluoride (**LiF**), (see Figure 1). Our PLEDs’ performance was compared against other electroluminescent materials reported in the same spectral region, finding that the turn-on voltage (*V*_on_) and the efficiency compare favorably. On the other hand, **PF–1** exhibits good lasing properties in solution. The laser threshold and laser tunability range of **PF–1** were measured and compared with the well-known laser dye rhodamine 6G and with other materials tested as gain media. Our results show that the cross-conjugated polymer **PF–1** is a promising candidate for PLEDs and lasing applications.

## 2. Materials and Methods

### 2.1. Materials

Synthesis of the fluorene derivative monomer 4,7-bis[2-(9,9-dimethyl)fluorenyl] benzo[1,2,5] thiadiazole and its cross-conjugated polymer (**PF–1**) were reported elsewhere [32]. The molecular structure of **PF–1** and the PLED architecture are shown in Figure 1. Linear absorption and lasing measurements were performed in solution with a concentration of 10^−4^ M using chlorobenzene (purchased from Sigma-Aldrich, Mexico), as solvent.

PLEDs were fabricated using an Indium Tin Oxide (**ITO**)-covered glass substrate ultrasonically cleaned for 30 min in each of the following baths: distilled water, ethanol and an alkaline solution (Hellmanex II mixed with water). These substrates were coated by spin-coating with **PEDOT:PSS** to create a hole injector film (40–50 nm thick) and annealed in air at 120 °C. The emitter layer was deposited from a solution of **PF–1** dissolved in chlorobenzene (6 mg/mL), and consisted of an 80–120 nm thick film covering 0.09 cm^2^ of active area. Deposition was done in a glove box under a nitrogen atmosphere. Once deposited, the organic films were annealed (in presence of air) at 120 °C for 20 min. Two different sets of PLEDs were prepared with the commonly used electron injectors **PFN** and **LiF**. The **LiF** layer was deposited by vacuum evaporation and had a thickness of 3 nm. It is worth noting that the typical **LiF** thickness for this type of device is between 0.5 and 2 nm [33,34]; however, our 3 nm **LiF** layer resulted after optimization of the PLED performance. For films below 3 nm, by using AFM analysis, we observed physical damage in the **PF–1** layer after deposition of the Aluminum (**Al**) cathode. **PFN** layer was deposited by spin-coating (≈10 nm of thickness) and annealed at 80 °C for 20 min. Finally, the **Al** cathode was deposited by thermal evaporation with a thickness of 100 nm. Morphological and film thickness measurements were performed by Atomic Force Microscopy (AFM) (Nanosurf, easyscan2, Woburn, MA, USA) operating in contact mode with a scanning area of 10 μm × 10 μm. For future comparison of surface roughness (Rα) presented in Section 3.1 between **PF–1** and poly[2-methoxy,5-(2’-ethylhexyloxy)-1,4-phenylene-vinylene] (**MEH-PPV**) films, according to [35]: “A 180 nm ITO film was grown. ITO glasses were cleaned by sequential ultrasonification in trichloroethylene, acetone, and methanol solvents for a total of 30 min. A thin layer of polyethylene dioxythiophene doped with polystyrene–sulfonic acid (PEDOT:PSS, Sigma-Aldrich) was spin-coated at 4000 rpm for 30 s on the cleaned ITO-coated glass substrate. Typical thicknesses of 70 nm resulted. The thin layers were then dried at 100 °C for 30 min. Spin-coating for photoactive layer deposition was kept at 3000 rpm to obtain a smoother surface with 150 nm active layer thickness. A 90 nm Al cathode layer was deposited on the active layer by thermal evaporation”.

### 2.2. Experimental

Steady-state linear absorption spectra were acquired, in solution, using a spectrophotometer (Perkin Elmer, Lambda 900, Waltham, MA, USA) over a range of 280 to 700 nm. Fluorescence emission curves were recorded with a portable spectrometer (Ocean Optics, USB4000, Dunedin, FL, USA); samples were illuminated with an UV light lamp source. The current density *versus* voltage (J–V) and luminous efficiency *versus* voltage (L–V) curves were measured simultaneously using a power supply (Newark element I4, Keithley 2400, Palatine, IL, USA) over a range of 0–14 V with an in-house-designed and calibrated detection system. The J–V curve is recorded by direct processing of data sent from the used Keithley 2400 apparatus. Luminous density is estimated through the voltage delivered by a photodiode located at fixed distance from the PLED. Photodiode calibration was performed by using the luminance of commercial LEDs, at different wavelengths and considering all geometrical parameters involved in the detection system. Signal was previously quantified by a highly sensitive lux meter and correlated with the photodiode voltage response. All data acquisition routines were automated by using LabVIEW software specially designed for this purpose. PLEDs emission was characterized under atmospheric conditions.

**Figure 2:**
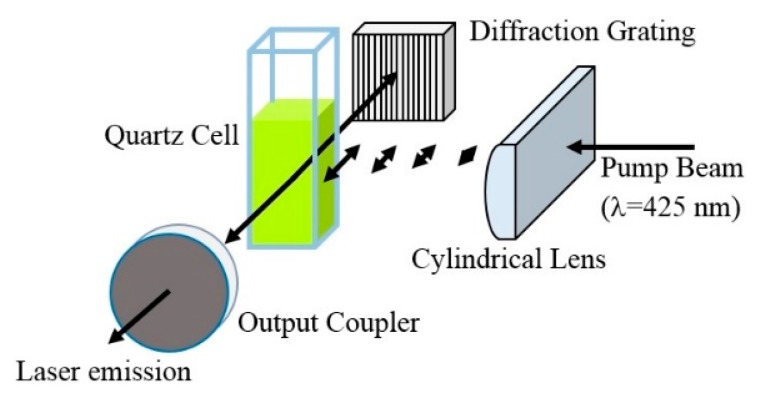
Experimental setup for lasing characterization.

For the **PF–1** laser emission studies, a 10 mm fluorometric quartz cuvette was placed in a flat nondispersive resonator and transversely pumped at 425 nm by an Optical Parametric Oscillator (OPO) system (Flexiscan, GWU Laser Technik, Germany) with maximum energy of 180 mJ and 6 ns pulse duration. The OPO was pumped by the third harmonic (355 nm) of a Nd:YAG laser (Spectra Physics, Quanta-Ray, Santa Clara, CA, USA) operating at 10 Hz in the nanosecond regime. The light beam from the OPO was focused by a cylindrical lens (focal distance 100 mm leading to spot line of area ~0.01 cm^2^) into a **PF–1** solution with 10^−4^ M concentration using chlorobenzene as solvent. The beam energy was varied using neutral density filters and the spectral width of the laser emission was determined by a spectrometer (Ocean Optics, USB4000, Dunedin, FL, USA) with a spectral resolution of 0.7 nm. Laser emission cross-section for stimulated emission was calculated according to reference [36]. In order to tune the emission wavelength of the **PF–1** solution, we replaced one of the resonator flat mirrors with a diffraction grating mounted on a kinematic stage. The experimental setup is shown in Figure 2.

Finally, fluorescence lifetime was obtained through time-correlated single photon counting (TCSPC) with a fluorescence lifetime system (Horiba, Tempro, Japan) equipment by using 370 nm nanoLEDs for excitation. **PF–1** sample was analyzed in chloroform solution (OD at 370 nm <0.1). A 0.01% suspension of Ludox AS40 (Sigma-Aldrich, Mexico) in ultrapure water was used for the prompt signal. Calibration of the equipment was performed with a [1,4-bis(4-methyl-5-fenil-2-oxazolyl)benzene] (**POPOP**) methanol solution (optical density <0.1 and lifetime of 0.93 ns [37]). Data were fit with the software DAS6 available in the equipment.

## 3. Results and Discussion

### 3.1. PLEDs

**Figure 3:**
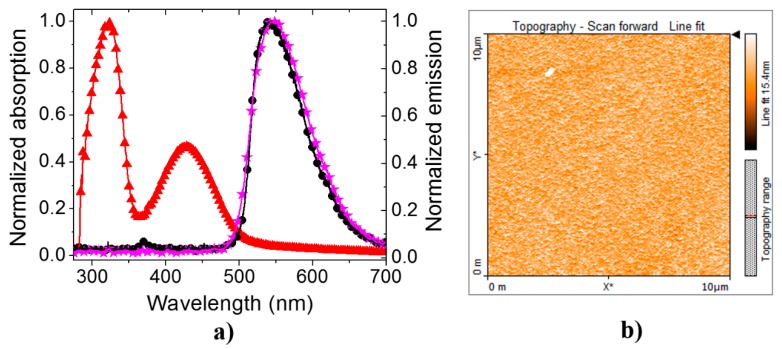
(**a**) Normalized absorption (red triangles), fluorescence emission (black circles), both in solution of chlorobenzene, and electroluminescence emission (purple stars) in solid state of **PF–1**; (**b**) AFM image of a **PF–1** thin film surface (*R*_α_ of the order of 1.4 nm).

Absorption and fluorescence spectra of **PF–1** in a solution of chlorobenzene and its electroluminescence spectrum in solid state are shown in Figure 3a. Here, two bands of maximum absorption are observed: a peak centered at 323 nm with a full width at half maximum (FWHM) close to 50 nm and a secondary peak with amplitude reaching almost 50% of the previous one, at 427 nm with a FWHM of approximately 75 nm.

The absorption peak centered at 323 nm is attributed to the π–π* transition of the conjugated chain. The second peak centered at 427 nm is associated with the n–π* transition of the benzothiadiazole, according to calculations presented in reference [29]. Both the steady-state fluorescence (black circles) and electroluminescence (purple stars) curves show emission peaks of **PF–1** in the green-yellow region with broad emission bands centered at 544 nm (FWHM of 80 nm) and 551 nm (FWHM of 90 nm), respectively. Fluorescence quantum yield in solution was determined to be close to one [31]. These results indicate that **PF–1** is able to recombine excitons efficiently; therefore, it is interesting to study the potential of the material for applications such as PLEDs or lasing.

**PF–1** solutions with different concentrations were prepared, observing a high solubility without aggregated molecules. Good solubility, among other properties of polymers, is important in order to process them into high quality thin films using wet processes, *i.e.*, spin-coating. For instance, the surface quality of the deposited films is critical for device performance since it influences the charge injection, mobility and recombination properties on the active film; it also influences the contact quality between the emitter layer and the hole and electron injector materials. Thus, this parameter could largely improve PLEDs’ emission efficiency. Figure 3b shows a surface topography image of the **PF–1** layer used for our PLEDs; the image covers an area of 10 μm × 10 μm. As seen in the figure, there exists a good surface roughness (*R*_α_) quality in our samples (*R*_α_ of the order of 1.4 nm). This number is in fact smaller than those typical values for the widely and previously used **MEH-PPV** polymer (~7–14 nm [35], deposited under very similar experimental conditions to those in this work, see Section 2.1), and **ITO** (~4–24 nm [38]).

**Figure 4:**
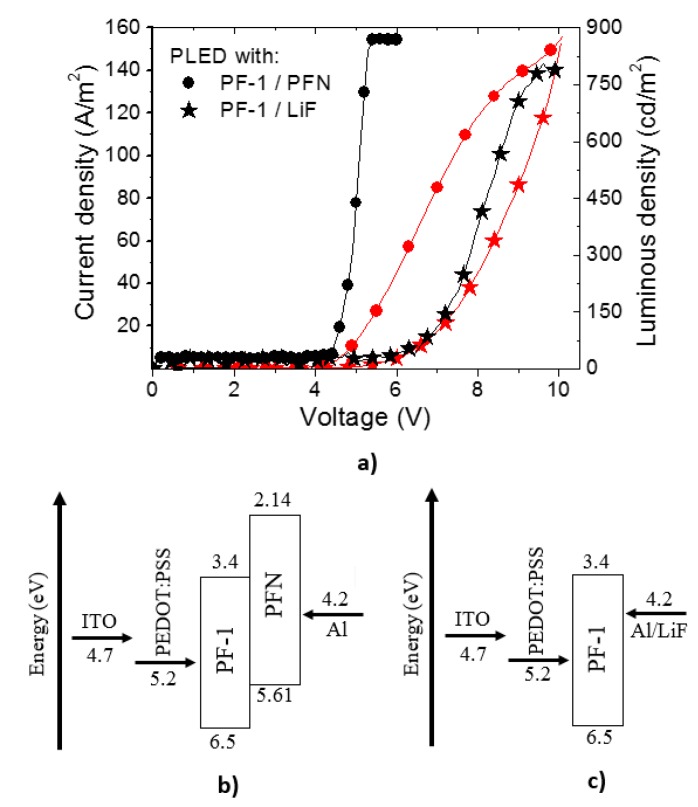
(**a**) Current density (red symbols) and luminous density (black symbols) as a function of applied voltage for PLEDs containing **PFN** (circles) and **LiF** (stars) as electron injectors, respectively. Schematic energy diagram for HOMO and LUMO levels for PLEDs with (**b**) **PFN** and (**c**) **LiF**.

Results from the measurements of current and luminosity as a function of the applied voltage for PLEDs comprising **PFN** or **LiF** as an electron injector layer are shown in Figure 4a. For the J–V curves, it is observed that the current increases over the range 4–10 V, reaching a maximum at approximately over 120 A/m^2^ for both devices. For PLEDs containing **PFN** (black circles), the luminous density increases sharply until 878 cd/m^2^ in the range 4.5–5 V. This increment is due to a lowering of the effective barrier height for the electron injection by **PFN** which leads to a more balanced injection of electrons and holes in the emitting layer [39]. **PFN** film creates a positive interface that induces a negative dipole potential for electrons, allowing their fast transfer between the **Al** metal cathode and the emitting polymer [39]. For voltages greater than approximately 5.5 V, the current density continues increasing although the luminous density seems to saturate, which could be due to limitations in the used photodetector; thus, it is probable that our reported PLED would be even more efficient. The energy level arrangement of the system is shown in Figure 4b. Here it is observed that **PFN** creates a potential barrier between the cathode and **PF–1**, allowing the transport of electrons by tunneling, and thus increasing the electron population in **PF–1**; this in turn leads to an increment of the luminous density of the device since **PF–1**, due to its light emission properties, can be cataloged as a good emitter material.

PLEDs based on **LiF** showed an increase in the luminous density reaching a maximum of 805 cd/m^2^ over a range between 6.5 to 9.5 V (see black stars in Figure 4a). Electrons are injected by tunneling over the potential barrier formed between the **Al** cathode and the emitter material, as shown in Figure 4c [40].

**Figure 5:**
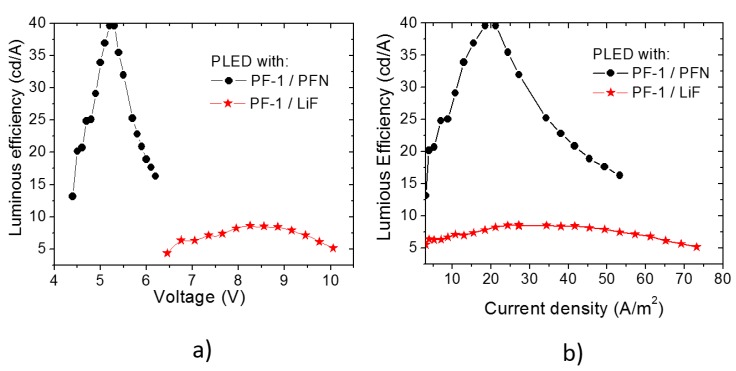
Luminous efficiency of PLEDs based on **PF–1** polymer with the electron injectors **PFN** and **LiF** as a function of (**a**) applied voltage, and (**b**) current density.

Results for the luminous efficiency of PLEDs containing **PFN** and **LiF** *versus* applied voltage and current density are shown in Figure 5a,b, respectively. It is seen that PLED**s** fabricated with **PFN** (black circles) (Figure 5a) have a turn-on voltage between 4 and 4.5 V and a maximum efficiency of 40 cd/A at 5.5 V. Similar behavior is displayed in Figure 5b where an increase in the efficiency is presented from 12.5 to 40 cd/A over a range of 0–20 A/m^2^, followed by a decrease in the efficiency to 17.5 cd/A which extends to 50 A/m^2^. In regards to the PLED**s** containing **LiF** (red stars in Figure 5a,b), the turn-on voltage was slightly over 6.5 V, and the maximum efficiency was around 9 cd/A, at 8 V (see Figure 5a). From Figure 5b, a low variation in efficiency is observed over the range between 5 to 70 A/m^2^, with its maximum at 30 A/m^2^. Both PLEDs’ architectures show a maximum luminous density above 800 cd/m^2^. This can be explained because the strongly polarizable central fragment and the “isatin” unit of **PF–1** allows electrons and holes to be transported efficiently along the polymer chain in order to be recombined. From our results, it is clear that PLEDs with **PFN** as the electron injection film show lower turn on-voltage and better efficiency than PLEDs containing **LiF**.

Our PLEDs compare favorably to other electroluminescent devices recently reported in the literature with similar emission wavelengths and architecture. Szlachcic *et al.* [41] achieved a luminous density of 600 cd/m^2^ at 8 V but they did not report the luminous efficiency; here, the helical fused azulene (**DDH**) as an emitter was used. Poloek *et al.* [42] obtained a luminous efficiency of 25.9 cd/A at 9 V with approximately 1000 cd/m^2^ with a heteroleptic platinum complex (**FPtppND**); Kumar Gupta *et al.* [43] reported a luminous efficiency of 1.71 cd/A at 10 V with a luminous density of 10 cd/m^2^ for a cross-linking polymer (**PIM–1**). Also, the efficiency compares favorably with that reported by Diken *et al.* [44] for much more complex copolymers containing carbazole and oxadiazole (PPV derivatives such as **MEH–PPV**) doped with a host triplet emitter material (carbazole homopolymer) with luminous efficiencies of 0.43, 15 and 23 cd/A. From this comparison, we conclude that our PLEDs can be considered as potential candidates for electroluminescence applications. Table 1 summarizes our results and some comparisons between different organic emitter materials.

**Table 1:** Parameters of **PF–1** PLEDs and their comparison with other organic electroluminescent materials with similar emission wavelengths.

Material	λ of Emission (nm)	Turn on voltage	Luminous density (cd/m^2^)	Efficiency (cd/A)	Ref.
PF–1 /PFN	551	4.5	878	40	This work
PF–1/LiF	551	6.5	805	9	This work
DDH	555	8	600	NA	[41]
FPtppND	545	9	1000	25.9	[42]
PIM-1	515	10	10	1.71	[43]
Carbazole homopolymer	527^a^	5	NA	0.45–15, 23^b^	[44]

^a^ Emission of triplet states; ^b^ Luminous efficiencies for different concentrations of **TPBI** exciton blocking.

### 3.2. Lasing of PF–1 Compound

**Figure 6:**
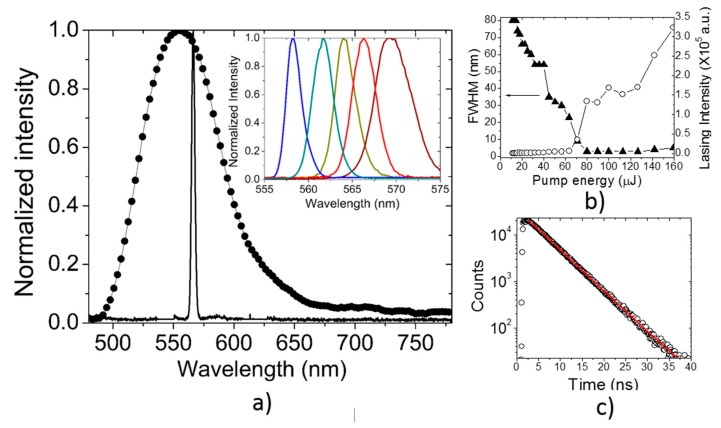
(**a**) Fluorescence (circles) and lasing (narrow line) of **PF–1** in chlorobenzene solution. Inset, tunability of **PF–1** lasing; (**b**) FWHM and lasing intensity of 566 nm emission for a solution of **PF–1** as a function of the pump energy; (**c**) **PF–1** fluorescence lifetime.

Most of the research in the field of polymer lasers focuses on creating materials with high optical gain, leading to a reduction of the laser threshold. As discussed before, the thermal and photochemical stability, high quantum yield of fluorescence and the possibility of being incorporated in solid-state matrices make **PF–1** a possible candidate for laser applications. Figure 6a shows the laser line emitted from a **PF–1** solution (10^−4^ M, in chlorobenzene) pumped at 10 Hz at the wavelength of 425 nm with pulses of 6 ns giving a peak power of 13 kW, focused on a spot line with an area of ~0.01 cm^2^, with a corresponding fluence and intensity of 7.8 mJ/cm^2^ and 1.3 MW/cm^2^. This light peak is centered at 566 nm with a FWHM of approximately 3.8 nm. To obtain the tunability range of the laser, the resonator flat mirror was replaced with a diffraction grating, finding tunability from 555 to 575 nm as shown in the inset of Figure 6a. Outside of this range, laser output was observed but its intensity was not constant. A laser threshold at 566 nm of around of 75 μJ (7.5 mJ/cm^2^) was found, as shown in Figure 6b. Also, Figure 6b shows two effects directly related to the pump energy; a constant reduction of the FWHM (filled triangles) of the emission band in the range 10–75 μJ; fluorescence; and a constant increase in the lasing intensity (open circles) in the range 75–160 μJ. The cross-section σ_e_ for the stimulated emission was calculated according to [36]:

(1) $$\sigma_{e}=\frac{\lambda_{e}^{4}E\left(\lambda \right)\varphi_{f}}{8\pi c_{0}n^{2}\tau_{f}}$$

where λ_e_ is the emission wavelength, *n* is the refractive index of the solvent, *c_o_* is the velocity of light, τ_*f*_ is the fluorescence lifetime, *E(*λ*)* is the normalized fluorescence line-shape function and ϕ_*f*_ is the quantum yield. Figure 6c shows the fluorescence decay of **PF–1** in a semi-log plot and fit to a line giving a lifetime τ_*f*_ of 4.9 ns. The calculated value of σ_e_ is 4.24 × 10^−16^ cm^2^, which is comparable with values reported recently for materials considered to be good laser dyes [36,37].

The laser threshold and the stimulated cross-section of **PF–1** compare well against the standard and widely used laser dye rhodamine 6G (laser threshold 70–75 μJ, intensity 4.4 MW/cm^2^, fluorescence lifetime of 3.9 ns, quantum yield 0.89, laser emission cross-section 4.17 × 10^−16^ cm^2^) [45,46,47,48]. **PF–1** as gain medium compares favorably to other materials recently reported in the literature. El-Daly *et al.* reported for dyes 3-(4-dimethylamino-phenyl)-1-(2,5-dimethyl-furan-3-yl)-propenone (**DDFP**) [36] and **POPOP** [37] fluorescence times of 2.3 and 0.93 ns and laser emission cross-section**s** of 3.23 × 10^−16^ cm^2^ and 2.82 × 10^−16^ cm^2^, respectively. Direct comparison of the lasing parameters is shown in Table 2. Regarding the lasing properties of **PF–1**, the higher cross-section of the stimulated emission and fluorescence lifetime compared with other materials (see Table 2) can be attributed to the large population of excited species associated with π–π* and *n*–π* transitions and the creation of electron–hole pairs located on different conjugated segments along the polymer chain. These electron–hole pairs can move along the polymer chain to reach the recombination length path causing longer-lived stimulated emission effects leading to extended fluorescence lifetimes [49,50,51,52]. In this regard, Pauck *et al.* [49] concluded that the amplitude as well as the lifetime of the stimulated emission is increased in diluted LPPP polymer blends with well-separated chains. A similar conclusion was stated by Yan *et al.* [50] and Rothberg *et al.* [51] for blends of soluble PPV. In contrast, optical pumping of laser dyes (such as rhodamine 6G, **POPOP** and **DDFP**) is usually achieved in high-lying vibrionic levels of the excited state, followed by an ultrafast vibrational relaxation causing an inversion of population and finally emission. In terms of laser physics the situation described above corresponds to a four-level system. From the mentioned comparisons and based in these attractive properties exhibited by **PF–1**, such as broad absorption and emission spectra, efficient photoluminescence emission and ease of processing, it is possible to position it as a potential candidate for laser applications.

**Table 2:** Laser emission cross-section (σ_e_), lifetime of fluorescence (τ_f_) and λ of maximum lasing for **PF–1** polymer and comparison with other organic materials used as laser gain media.

Material	Solvent	λ of maximum lasing (nm)	σ_e_ × 10^−16^ cm^2^	τ_f_ (ns)	Fluorescence range (nm)	Ref.
PF1	Clorobnezene	566	4.24	4.9	520–600	This work
Rhodamine 6G	Ethanol	566	4.17	3.9^a^	530–590	[46,47]
POPOP	Methanol	555	2.82	0.93	400–460	[36]
DDFP	DMSO	515	3.23	2.3	470–535	[37]

^a^ in methanol solution.

## 4. Conclusions

Light emission properties of **PF–1** under electrical and optical excitation have been studied. We implemented PLEDs based on **PF–1** polymer with two widely used electron injectors, **PFN** and **LiF**, in order to improve the performance and to compare them with devices emitting in the same spectral region reported by other groups. The following parameters were measured: for PLEDs with **PFN** as the electron injector layer, a V_on_ ~ 4.5 V, luminance of 878 cd/m^2^ at 5.5 V and 20 A/m^2^; and for PLEDs with **LiF**, V_on_~6.5 V, luminance of 805 cd/m^2^ at 9.5 V and 40 A/m^2^. In the case of **PFN** a very acceptable luminous efficiency at low current density and on-voltage have been reached. Meanwhile, PLEDs that contained **LiF** showed a luminous efficiency that remained approximately constant during a range of current density between 0 and 75 A/m^2^. It is concluded that **PF–1** is a good emitter material that favors an efficient transport of electrons and holes in order to be recombined. Lasing properties measured for **PF–1** found a low threshold (around 75 μJ, fluence and intensity of 7.5 mJ/cm^2^ and 1.25 MWcm^2^, respectively) for laser emission and a tunability of 25 nm. These values are reasonable in comparison with those of the standard laser dye rhodamine 6G. These light emission studies of **PF–1** show a robust cross-conjugated polymer and position it as a good candidate for electroluminescent devices as well as for possible implementation in laser applications.
